# A novel TRKB-activating internal tandem duplication characterizes a new mechanism of receptor tyrosine kinase activation

**DOI:** 10.1038/s41698-025-00928-3

**Published:** 2025-05-10

**Authors:** Lauren M. Brown, Gabor Tax, Pablo Acera Mateos, Antoine de Weck, Steve Foresto, Thomas Robertson, Fatimah Jalud, Pamela Ajuyah, Paulette Barahona, Jie Mao, M. Emmy M. Dolman, Marie Wong, Chelsea Mayoh, Mark J. Cowley, Loretta M. S. Lau, Teresa Sadras, Paul G. Ekert

**Affiliations:** 1 Children’s Cancer Institute, Lowy Cancer Research Centre, UNSW Sydney, Sydney, NSW Australia; 2 School of Clinical Medicine, UNSW Sydney, Sydney, NSW Australia; 3 Queensland Children’s Hospital, Brisbane, QLD Australia; 4 Peter MacCallum Cancer Centre, Parkville, VIC Australia; 5 The Sir Peter MacCallum Department of Oncology, University of Melbourne, Parkville, VIC Australia; 6 Kids Cancer Centre, Sydney Children’s Hospital, Randwick, NSW Australia; 7 University of New South Wales Centre for Childhood Cancer Research, UNSW Sydney, Sydney, NSW Australia

**Keywords:** Paediatric cancer, Oncogenes, Growth factor signalling, Cancer genomics

## Abstract

Precision medicine programs like the Zero Childhood Cancer Program perform comprehensive molecular analysis of patient tumors, enabling detection of novel structural variants that may be cryptic to standard techniques. Identification of these variants can impact individual patient treatment, and beyond this establish new mechanisms of oncogenic activation. We have identified a novel internal tandem duplication (ITD) in the receptor tyrosine kinase (RTK), *NTRK2*, in a patient with FOXR2-activated CNS neuroblastoma. The ITD spans exons 10-13 of *NTRK2* encoding the transmembrane domain. NTRK2 ITD is transforming and sensitive to TRK inhibition. In silico structural predictions suggested the duplication of an alpha-helix region and juxtaposed tyrosine residues that play a role in facilitating autophosphorylation. Consistent with this, mutation of these residues inhibited cellular transformation. This is the first report of an ITD spanning the transmembrane domain of an RTK, characterizing an additional mechanism by which RTKs are activated in cancer.

## Introduction

Precision medicine programs like the Zero Childhood Cancer Program (ZERO) are changing the prognostic outlook for pediatric cancer by using comprehensive sequencing approaches to identify additional therapeutic options for patients^1^. The advantage this has over standard of care cytogenetics and panel sequencing is the capacity to identify structural variants (SVs) and mutations beyond the spectrum that is established for a particular tumor type, including novel gene fusions and intragenic deletions that are often cryptic to standard techniques.

Analysis of long-term clinical outcome data of patients enrolled in ZERO has demonstrated that molecularly targeted treatments can improve outcomes (26% improved 2-year progression free survival vs. 12% for standard of care)^2^. Targeted treatments were most effective when administered early (before disease progression), highlighting the need to not only identify these lesions in the first instance but to also understand the therapeutic implications of the specific lesion. Targeting tyrosine kinases (TKs), including receptor tyrosine kinases (RTKs), has been particularly effective in pediatric cancers in the context of bona fide activating events, such as the *BCR::ABL1* fusion gene, and as such, identifying alterations in TK genes remains a high-priority in precision medicine^3^.

The TRK family kinases (encoded by *NTRK1*, *NTRK2*, and *NTRK3*) are activated in cancer by a range of mechanisms, including gene amplification, mutation, and gene fusions. In pediatric cancers, *NTRK* fusions are enriched in rare tumor types, including infantile fibrosarcoma and infant high-grade glioma, and are sensitive to TRK inhibition^4^. Given the remarkable clinical success of TRK inhibitors, the question arises about whether the use of these inhibitors could be expanded to other NTRK2 aberrations. We have identified a novel internal tandem duplication (ITD) in *NTRK2*, spanning the juxtamembrane and transmembrane protein domains in a pediatric patient diagnosed with Forkhead Box R2 (FOXR2)-activated CNS neuroblastoma. Here, we present the functional characterization of NTRK2 ITD as a TRKB-activating variant that is sensitive to TRK inhibition. Drug screening revealed unique therapeutic vulnerabilities of NTRK2 ITD, compared to the established SPECC1L::NTRK2 fusion^5^, and in silico analysis suggests an alternate mechanism of activation whereby duplication of the transmembrane alpha-helix and acquisition of additional tyrosine phosphorylation sites may play a role in transformation.

## Results

### WGS identifies a novel NTRK2 ITD resulting in duplication of the transmembrane domain

**Fig. 1:**
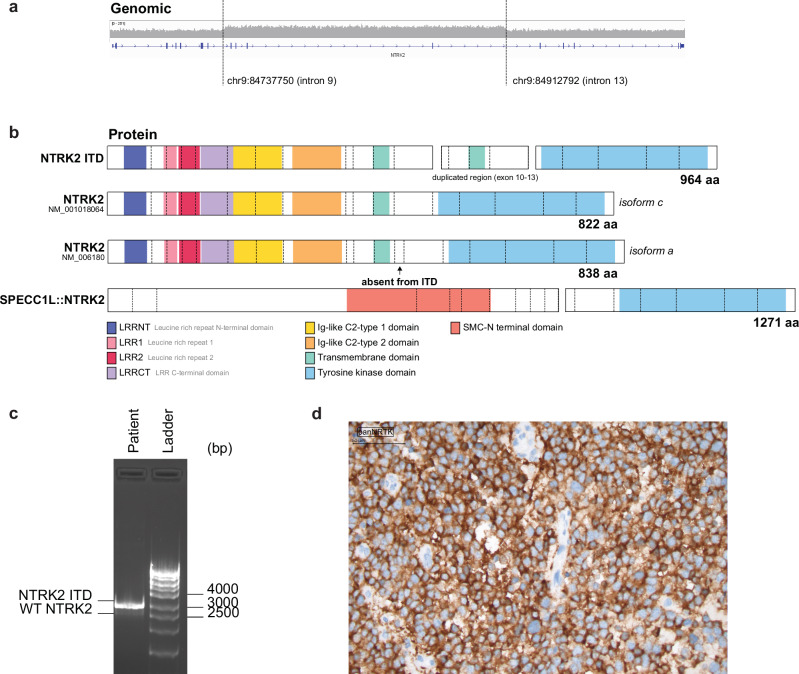
WGS identifies a novel NTRK2 ITD resulting in a duplication of the transmembrane domain. **a** WGS identifies a 175 kb duplication in chromosome 9, spanning intron 9-13 of *NTRK2* (NM_001018064) in a patient diagnosed with CNS neuroblastoma. **b** NTRK2 ITD results in an in-frame fusion transcript that encodes a 964 amino acid protein with duplication of exon 10-13, including the transmembrane domain. Schematics were generated with ProteinPaint^23^ and show NTRK2 ITD, two isoforms of WT NTRK2, isoform c and isoform a, and the SPECC1L::NTRK2 gene fusion, an established kinase-activating variant. **c** PCR performed on patient cDNA with primers targeting the 5’ and 3’ ends of *NTRK2* amplified NTRK2 ITD and WT NTRK2. **d** Immunohistochemistry staining of patient tumor tissue with an anti-Trk antibody.

We identified a novel ITD involving *NTRK2* in a 5-year-old girl diagnosed with FOXR2-activated CNS neuroblastoma (see Methods for clinical case description). The duplication event spanned intron 9-13 (hg38: chr9:84737750-84912792) of *NTRK2* and was initially missed by RNAseq-based gene fusion and SV detection tools (Fig. 1a). While RNAseq-based variant detection tools are not optimized to detect ITDs that span multiple exons, this NTRK2 ITD was confirmed at the transcriptome level through manual inspection of RNAseq reads as a duplication of exons 10-13 of *NTRK2* isoform c (NM_001018024). This transcript was predicted to encode a TRKB protein with duplication of a 141 amino acid region (Ala388-Thr528) spanning the transmembrane domain (Fig. 1b and Supplementary Fig. 1). PCR amplification from patient cDNA across the duplicated region confirmed the presence of *NTRK2 ITD* and wild type (WT) *NTRK2* transcript (Fig. 1c and Supplementary Fig. 2) and pan-TRK immunochemistry (IHC) confirmed high-intensity extranuclear staining in the tumor (Fig. 1d), compared to control staining (normal brain, Supplementary Fig. 3). This tumour had low *BDNF* RNA expression, encoding the ligand for TRKB.

### NTRK2 ITD is constitutively activated

**Fig. 2:**
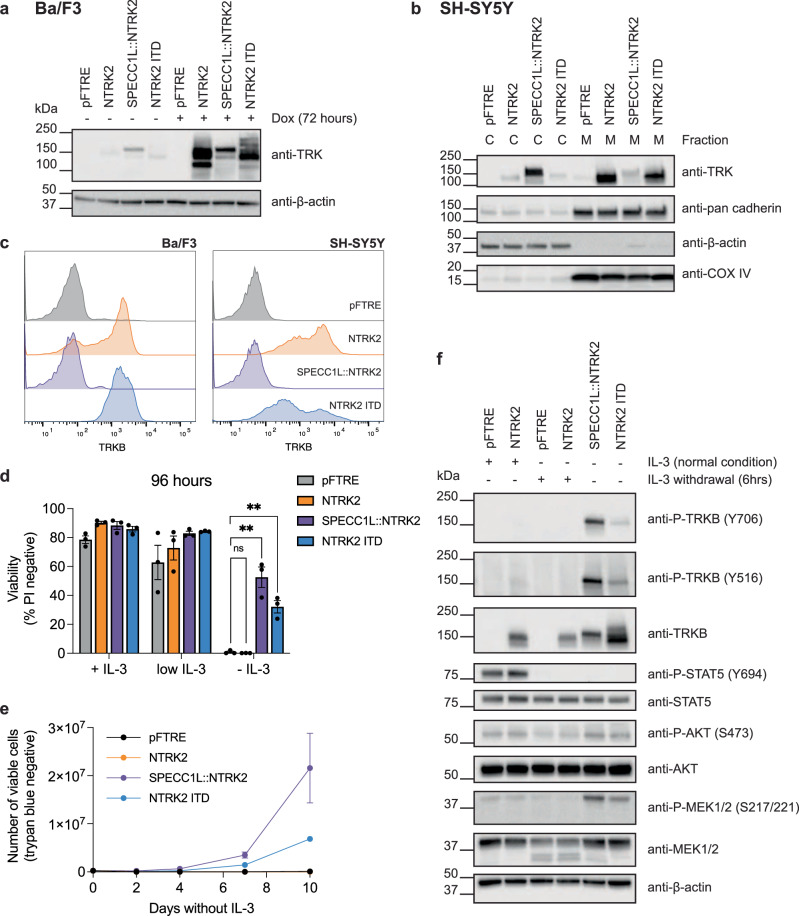
NTRK2 ITD is constitutively activated. **a** Western blot analysis of Ba/F3 cells with doxycycline (dox) inducible expression of NTRK2, SPECC1L::NTRK2, and NTRK2 ITD, or empty vector control (pFTRE). **b** Western blot analysis of cytoplasmic (C) and membrane (M) protein fractions derived from SH-SY5Y cells with dox-induced expression of NTRK2. β-actin, pan-cadherin, and COX IV were used as cytoplasmic, plasma membrane, and mitochondrial membrane markers, respectively. **c** Cell surface expression of TRKB in Ba/F3 and SH-SY5Y cells transduced with NTRK2 variants, determined by cell surface staining with an anti-Trk antibody and flow cytometry analysis. These plots are representative of two biologically independent repeats in each cell line (*n* = 2). **d** Viability analysis of Ba/F3 cells cultured in normal conditions (+IL-3), low IL-3 conditions, or no IL-3 conditions (-IL-3) for 96 hours. Viability was determined by PI exclusion measured by flow cytometry. NTRK2 variant cell lines were compared to empty vector control using unpaired *t* tests with Bonferroni-Dunn correction for multiple comparisons (ns = not significant, **P value ≤0.01). Data is presented as mean ± SEM (*n* = 3). **e** Number of viable (determined by trypan blue exclusion) Ba/F3 cells cultured without IL-3 over a 10-day period. Data is presented as mean ± SEM (*n* = 3). **f** Analysis of TRKB and downstream signaling pathway activation in Ba/F3 cells using Western blot analysis. Analysis was performed on pFTRE and NTRK2 Ba/F3 cells in either the presence of IL-3 or following 6-hour IL-3 withdrawal and transformed SPECC1L::NTRK2 and NTRK2 ITD Ba/F3 cells (no IL-3). Western blot is a representative image of three biologically independent repeats (*n* = 3).

Given the efficacy of TRK inhibitors to block oncogenic signaling from NTRK2 gene fusions^5^, we sought to establish if NTRK2 ITD is also TRK activating and sensitive to TRK inhibition. We used interleukin-3-(IL-3) dependent Ba/F3 cells as a robust cell line model to assess the transforming capacity of kinase-activating variants^6–8^. NTRK2 ITD was cloned in a doxycycline-inducible lentiviral vector (pFTRE) and expressed in Ba/F3 cells. WT NTRK2 and SPECC1L::NTRK2 were used as controls (Fig. 2a and Supplementary Fig. 4). Western blot analysis of cytosolic and membrane fractions isolated from Ba/F3 cells (Supplementary Fig. 5a) and the neuroblastoma cell line, SH-SY5Y (Fig. 2b and Supplementary Fig. 5b), showed that NTRK2 ITD expression was restricted to the membrane fraction. Further analysis of subcellular localization, through cell surface staining of Ba/F3 and SH-SY5Y cells (Fig. 2c and Supplementary Fig. 5c), with an anti-TRKB antibody directed to the extracellular region of TRKB, confirmed that NTRK2 ITD is expressed on the cell surface and localized to the plasma membrane. Expression of NTRK2 ITD promoted IL-3 independent cell survival (Fig. 2d) and proliferation (Fig. 2e and Supplementary Fig. 6), although transformation was delayed compared to SPECC1L::NTRK2. Addition of TRKB ligand, BDNF, was sufficient to overcome IL-3 withdrawal induced cell death in Ba/F3 cells expressing WT NTRK2 and promoted transformation (Supplementary Fig. 7a-b). In contrast, BDNF addition had no significant effect on accelerating transformation of NTRK2 ITD or SPECC1L::NTRK2 Ba/F3 cells (Supplementary Fig. 7a-b) and did not increase proliferation of SH-SY5Y cells expressing either SPECC1L::NTRK2 or NTRK2 ITD (Supplementary Fig. 7c). Western blot analysis of Ba/F3 cells showed that TRKB was phosphorylated in NTRK2 ITD-expressing cells and associated with downstream AKT and MEK phosphorylation (Fig. 2f and Supplementary Fig. 8). Taken together, these data show that NTRK2 ITD is localized to the plasma membrane, is TRKB-activating in a ligand-independent manner and potentially represents a novel oncogenic driver variant.

### NTRK2 ITD-expressing cells are sensitive to TRK and MEK inhibition

**Fig. 3:**
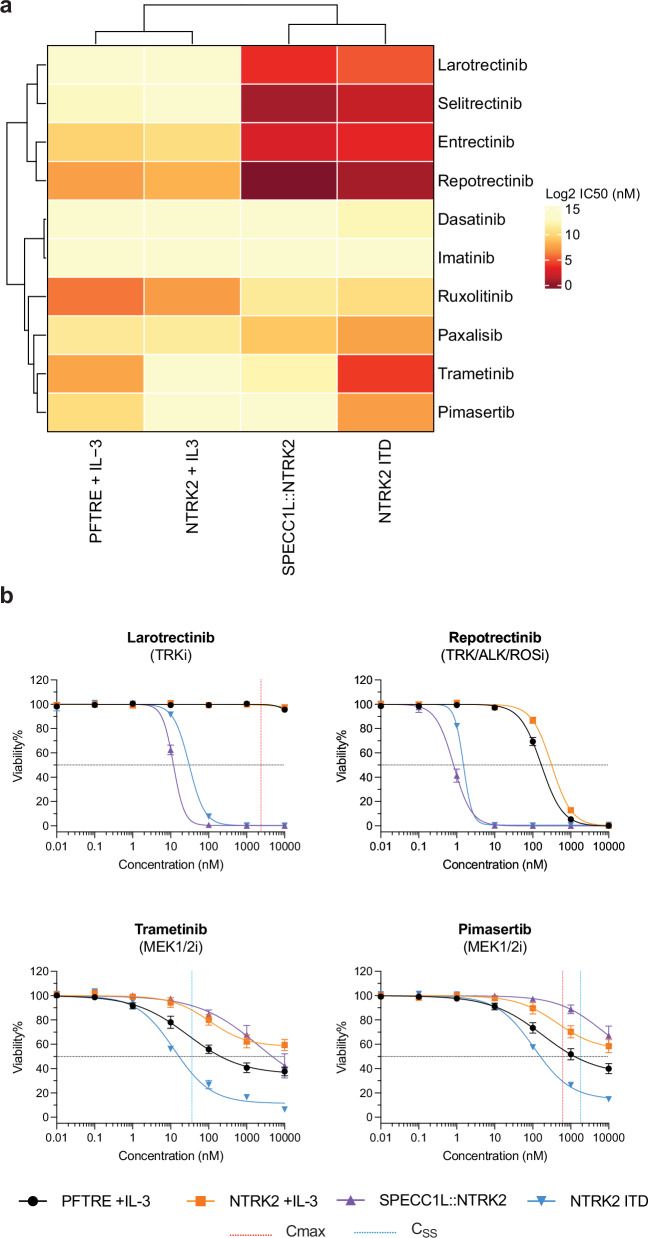
NTRK2 ITD-expressing cells are sensitive to TRK and MEK inhibition. **a** Heatmap displaying Log2 IC50 values (nM) determined for a range of kinase inhibitors used to treat Ba/F3 cells expressing NTRK2 variants or empty vector control (*n* = 3). **b** Dose response curves of Ba/F3 cells treated with TRK inhibitors, larotrectinib and repotrectinib, or MEK1/2 inhibitors, trametinib and pimasertib. Maximum serum concentration (Cmax) and steady-state concentration (C_ss_) of each of the drugs, if known, is represented by the orange and red dotted lines, respectively. IC50 values are depicted by the black dotted line. Cells were screened in technical triplicates in three biologically independent experiments and data is presented as mean ± SEM (*n* = 3).

We next tested sensitivity to TRK inhibition. We treated Ba/F3 cells transformed with NTRK2 ITD, or SPECC1L::NTRK2, with a panel of TRK inhibitors, as well as drugs targeting downstream signaling pathways, JAK/STAT, PI3K/AKT and MAPK. Ba/F3 cells expressing an empty vector (pFTRE) and WT NTRK2 were used as controls and were maintained in IL-3 for the screen. Like SPECC1L::NTRK2, NTRK2 ITD-expressing Ba/F3 cells were exquisitely sensitive to all four TRK inhibitors tested, with repotrectinib being the most effective (IC50 = 1.496 nM; Fig. 3a, Supplementary Fig. 9 and Supplementary Table 1 for IC50 values calculated for each drug). Sensitivity to TRK inhibition was also demonstrated in SH-SY5Y cells^9^, where cells expressing either NTRK2 ITD or SPECC1L::NTRK2 were equally sensitive to TRK inhibition in the presence and absence of BDNF, while BDNF addition increased the sensitivity of cells expressing WT NTRK2 (Supplementary Fig. 10). Both SPECC1L::NTRK2 and NTRK2 ITD-expressing cells were sensitive to the dual PI3K/mTOR inhibitor, paxalisib, with NTRK2 ITD-expressing cells being more sensitive (IC50 = 184.3 nM vs. 574.1 nM; Supplementary Fig. 9). Interestingly, NTRK2 ITD-expressing cells were specifically sensitive to MEK inhibition, compared to SPECC1L::NTRK2-expressing cells (trametinib IC50 = 12.33 nM vs. 2028 nM; Fig. 3b). This increased sensitivity of NTRK2 ITD-expressing cells suggests an increased dependency on activated MEK signaling for oncogenic cell survival.

#### The tyrosine residues within the duplicated region of NTRK2 ITD contribute to activation

**Fig. 4:**
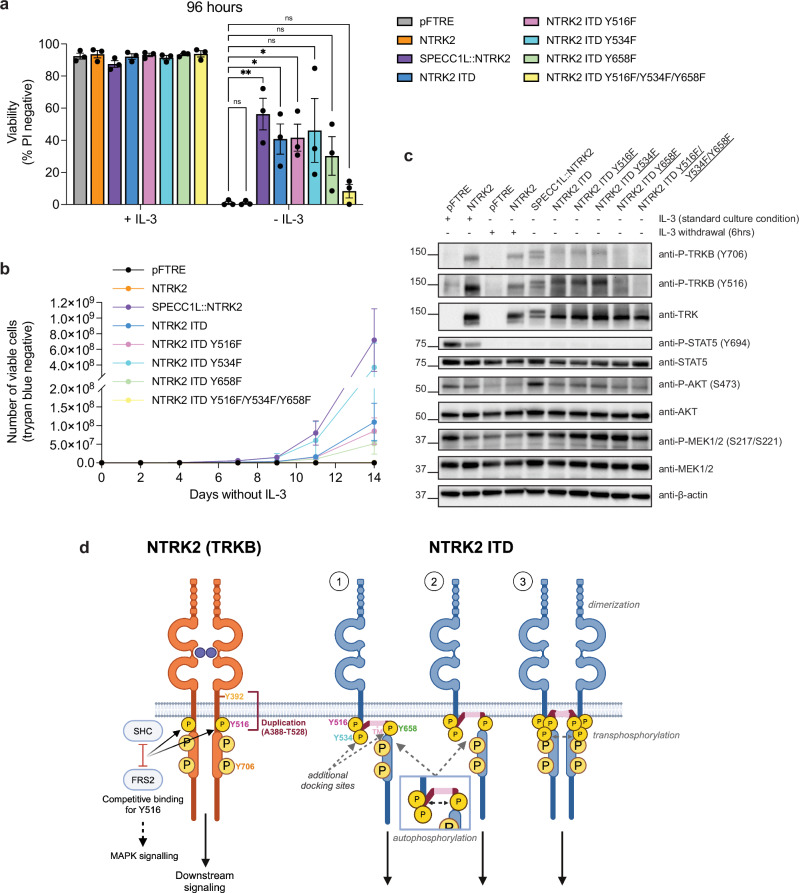
The tyrosine residues within the duplicated region of NTRK2 ITD contribute to activation. **a** Viability analysis of Ba/F3 cells transduced with NTRK2 ITD variants, Y516F, Y534F, Y658F, and Y516F/Y534F/Y658F (and experimental controls), cultured in normal conditions (+IL-3) and no IL-3 conditions (-IL-3) for 96 hours. Viability was determined by PI exclusion measured by flow cytometry. NTRK2 variant cell lines were compared to empty vector control using unpaired *t* tests with Bonferroni-Dunn correction for multiple comparisons (ns = not significant, *P value ≤0.05, **P value ≤0.01). Data is presented as mean ± SEM (*n* = 3). **b** Number of viable (determined by trypan blue exclusion) Ba/F3 cells expressing NTRK2 ITD variants cultured without IL-3 over a 14-day period. Data is presented as mean ± SEM (*n* = 3). **c** Western blot analysis of TRKB and downstream signaling pathway activation in Ba/F3 cells. Analysis was performed on pFTRE and NTRK2 Ba/F3 cells in either the presence of IL-3 or following 6-hour IL-3 withdrawal and on transformed cell lines for all other lines (no IL-3). Western blot is a representative image of three biologically independent repeats (*n* = 3). **d** Schematic of WT NTRK2 (TRKB) activation and the proposed mechanism for NTRK2 ITD activation. NTRK2 activation is normally mediated by ligand binding and receptor dimerization resulting in transphosphorylation and kinase activation. We propose that NTRK2 ITD retains the typical structure of WT NTRK2 with the addition of a transmembrane domain (TM, shown in pink) that most likely projects intracellularly (1) or spans the membrane into the cytoplasmic surface (2) and addition the presence of additional tyrosine sites (Y534 and Y658) may mediate autophosphorylation of NTRK2 ITD monomers. Alternatively, the duplicated alpha-helix transmembrane domain may mediate ligand-independent dimerization and transphosphorylation of NTRK2 ITD monomers (3). In addition to promoting autophosphorylation/transphosphorylation of the receptor, the acquired tyrosine residues may provide additional docking sites for signaling proteins, such as SHC/FRS2 which normally bind to Y516 and promote MAPK signaling activation, contributing to transformation. Created in BioRender. Brown, L. (2025) https://BioRender.com/r36g092.

We next considered how the duplicated region could affect signal transduction. MAPK and PI3K signaling activation is typically mediated by the binding of adaptor proteins, fibroblast growth factor receptor 2 (FRS2) and SHC-transforming protein (SHC), to phosphorylated tyrosine (Y516) at a conserved binding motif NPXp(Y) in TRKB^10^. NTRK2 ITD contains two additional NPXp(Y) motifs, Y534 (duplication of Y392 which is normally extracellular in WT TRKB) and Y658 (duplication of the canonical Y516 residue). To test the role these additional Y residues play in NTRK2 ITD signaling, we generated non-phosphorylatable Y to phenylalanine (Y > F) NTRK2 ITD mutant constructs of each residue (Y516F, Y534F, Y658F) individually, or combined (Supplementary Fig. 11a). While mutation of each individual residue did not affect transformation, mutation of all three either significantly delayed or completely abolished transformation by NTRK2 ITD (Fig. 4a, b and Supplementary Fig. 11b). Cell surface staining of transformed cells confirmed that Y > F mutations did not alter the localization of the protein from the plasma membrane (Supplementary Figs. 11c and 12a). Western blot analysis of NTRK2 ITD Y > F mutant cells showed that TRKB, AKT and MEK phosphorylation was not impacted by mutation of single Y residues (Fig. 4c and Supplementary Figs. 11d and 12b). However, mutation of all three Y residues abolished TRKB phosphorylation, even when cells were rendered IL-3 independent. In these cells, TRKB was not phosphorylated but downstream signaling was activated, suggesting there is an NTRK2 ITD-independent mechanism causing transformation. These data suggest that phosphorylation of duplicated Y residues (Y534 and Y658) is likely required for transformation by NTRK2 ITD.

We used in silico modelling tools (ESMfold^11^ and US-align^12^) to predict and compare the predicted protein structures of NTRK2 ITD to WT NTRK2. As predicted the NTRK2 ITD contains a second alpha helix domain (Supplementary Fig. 13), corresponding to the transmembrane domain of NTRK2. This duplicated alpha helix is flanked two disordered regions that contain the additional Y residues (Y534 and Y658). ESMfold has limited capacity to predict the structure of disordered regions, therefore the spatial distribution of these Y residues remains speculative. However, since NTRK2 ITD is membrane bound and activates intracellular TRKB signaling, it is likely that NTRK2 ITD retains the typical transmembrane protein structure of TRKB with an extracellular region and intracellular kinase domain, anchored by the canonical TRKB transmembrane domain. The localization of the alpha-helix, whether it bypasses the cell membrane or is intracellular, and the acquired Y residues is unclear (Fig. 4d).

## Discussion

Improving therapeutic outcomes from targeted therapies is dependent not only on the development of new targeted agents but also on understanding the range of variants against which they are effective. The novel TRKB activating variant, NTRK2 ITD we have described, is sensitive to existing TRK inhibitors. Our functional analysis revealed that NTRK2 ITD mediates a distinct mechanism of TRKB activation, compared to NTRK2 gene fusions, that is associated with an acquired sensitivity to MEK inhibitors.

The identification of other oncogenic ITDs in pediatric cancer (e.g. FLT3 ITD in AML^13^ and FGFR1 ITD^14^ in low-grade glioma) has typically relied on PCR-based assays. This approach requires prior knowledge of the ITD of interest and has no capacity to detect novel ITD variants. This is a clear advantage to using WGS, which should detect all small structural variants, including ITDs, that are often cryptic to other approaches^15^. The WGS approach in ZERO is not replicated in many pediatric precision medicine programs^16^, and so is not widely available. It is plausible therefore that other cases of NTRK2 ITD or ITDs spanning the transmembrane domains of other RTK genes have gone undetected, although the true incidence of these SVs is unknown, albeit still rare.

*NTRK* gene fusions have been identified in a diverse range of pediatric (notably infantile fibrosarcoma and high-grade glioma) and adult tumors, and in which TRK inhibitors can result in prolonged clinical response and improved patient outcomes^4,17^. NTRK alterations, including NTRK fusions have not been previously described in FOXR2-activated CNS neuroblastoma, although this is a relatively rare subtype of disease with molecular analysis limited to small cohorts^18^. Many *NTRK* gene fusions consist of a 5’ gene partner encoding a protein dimerization domain (e.g. coiled coil domain) fused to the tyrosine kinase domain of *NTRK*. However, other reported *NTRK* fusions do not adhere to this general structure and the mechanism of TRK activation in these contexts is unknown^19^. ITDs in *NTRK* genes or involving the transmembrane domain of other RTK genes have not been previously described and this study characterizes a novel mechanism of RTK activation that could be relevant for the interpretation of similar variants as they are identified.

NTRK2 ITD results in duplication of the alpha-helix transmembrane domain and the acquisition of two binding motifs that when phosphorylated at Y534 and Y658 play a role in cellular transformation. Mutation of Y534, Y658 and the canonical SHC/FRS2 binding site Y516 together significantly diminished, and likely abolished, transformation by NTRK2 ITD. Further, NTRK2 ITD-transformed Ba/F3 cells show increased sensitivity to downstream pathway inhibitors, mainly MEK inhibitors. These results suggest the possibility that in addition to activation of the TRKB kinase domain, NTRK2 ITD acts as a scaffold for downstream signaling activation, which may explain an increased dependency on MEK signaling supported by our drug screening data. TRKB is anchored to the membrane by a single-pass transmembrane domain^19^. The acquisition of a second transmembrane domain poses intriguing questions as to the true structure of the active ITD form. The mechanisms that govern membrane integration of hydrophobic alpha-helices are complex and can be influenced by levels of hydrophobicity, upstream protein sequences and protein orientational preferences^20,21^. Our data suggests that in the case of NTRK2 ITD, the canonical extracellular region of NTRK2 is expressed on the cell surface and the drug response data showed that NTRK2 ITD cells are dependent on TRKB signaling for survival.

Since *NTRK2* is not expressed in Ba/F3 cells, signaling activation cannot be through dimerization with WT NTRK2 in this model. We speculate that NTRK2 ITD likely retains the typical structure of WT NTRK2, with an additional alpha helix that either spans the membrane into the cytoplasmic surface or projects intracellularly. In either case, the duplicated alpha-helix may mediate ligand-independent dimerization of NTRK2 ITD monomers. Crucially, whatever the mechanisms, NTRK2 ITDs are an SV whose detection is important since existing drugs may be effective in treating tumors in which they are expressed. Moreover, the principals governing NTRK2 ITD activation may apply to transmembrane ITDs in other RTKs and constitute a novel class of targetable RTK-activating variants.

## Methods

### Clinical case description

A 5-year-old previously healthy girl presented to hospital in status epilepticus. Imaging demonstrated a solitary, predominantly solid mass centered in the right anteromedial middle cranial fossa, with intraparenchymal extension into the right temporal lobe. There was local mass effect including mild uncal herniation and midline shift of the septum pellucidum. There was no hydrocephalus and no evidence of leptomeningeal disease. The patient underwent a right sided temporal craniotomy and complete resection of the tumor was achieved. Post operative imaging demonstrated no evidence of disease. CSF cytology for staging was negative.

Histopathology showed an embryonal tumor composed of neuroblastic cells. Mitotic and apoptotic figures were easily found but there was no necrosis. Tumor was seen to infiltrate adjacent brain. Immunohistochemistry showed that the tumor expressed CD56, synaptophysin, Olig2, SOX10, TTF1/NKX2.1 and there was patchy immunoreactivity for NeuN and NSE. The tumor was negative for vimentin, NFP and GFAP. Normal nuclear staining for H3 K27me3 was preserved. The ki67 proliferation fraction was approximately 50% of tumor cell nuclei. The morphology and immunophenotype were consistent with a CNS neuroblastoma, FOXR2-activated (CNS WHO grade 4). ZERO molecular analysis identified a *TXLNG::FOXR2* structural rearrangement leading to elevated *FOXR2* expression. Tumor methylation profiling using the MNP classifier was a match to CNS neuroblastoma, FOXR2-altered (0.99) (v12.3), confirming this diagnosis.

The patient subsequently underwent adjuvant radiation consisting of 36 Gy VMAT craniospinal irradiation with primary site boost to the right temporal lobe. This was followed by four, 28-day cycles of chemotherapy consisting of vincristine, cisplatin and cyclophosphamide. At 2-years post-therapy the patient remains well and in complete remission.

### Identification and PCR validation of NTRK2 ITD

NTRK2 ITD was identified in this patient by whole genome sequencing (WGS) performed as part of ZERO. Methods for sample collection, sequencing, and data analysis have been previously described^1^. NTRK2 ITD expression was subsequently validated by PCR amplification from patient cDNA. Patient RNA was first reverse transcribed using the Invitrogen™ SuperScript™ III First-Strand Synthesis System (Thermo Fisher Scientific, catalogue number: 18080051). The duplicated region of NTRK2 was PCR amplified using Q5® Hot Start High-Fidelity DNA Polymerase (New England Biolabs, catalogue number: M0493) from patient cDNA using the following primers: NTRK2 exon 9 forward: 5’ GATTTCTGCTCACTTCATGGGC and NTRK2 exon 16/17 reverse: 5’ GTGCCCTGAGGAACTTGTTGAG. Primers targeting the 5’ and 3’ end of NTRK2 were designed to amplify and clone full length NTRK2 ITD and wild type (WT) NTRK2 as follows: EcoRI NTRK2 forward: 5’ AAGAATTCATGTCGTCCTGGATAAGG and NheI NTRK2 reverse: 5’ AAGCTAGCCTAGCCTAGAATGTCCAGG. Isolation of NTRK2 ITD was difficult given the co-expression of WT NTRK2, which is of a similar size. The ITD was PCR amplified using GoTaq® Long PCR Master Mix (Promega, catalogue number: M4021) and gel extracted for sequencing, which confirmed the sequence of the ITD. However, given the lack of proofreading activity in this DNA polymerase, the sequence had multiple mutations, which when correlated with patient WGS and RNA sequencing (RNAseq) data were confirmed to be absent from the patient. As such, the ITD was commercially synthesized by Twist Biosciences to correct these mutations (see DNA constructs).

### Immunohistochemistry

Immunohistochemistry staining for TRK was performed on the patient’s tumor sample by the Queensland Children’s Hospital Anatomical Pathology department. Staining was performed using the VENTANA® pan-TRK (EPR17341) Assay (Roche Diagnostics, catalogue number: 790-7026).

### Ethics and patient samples

This study was approved by the University of New South Wales Human Research Ethics Committee (Number: HC190443). Access to patient samples was approved and obtained through the ZERO Childhood Cancer Program Research Management Committee. Written informed consent, including consent for publication, for patients enrolled in the ZERO Program were provided either by the parent or legal guardian for patients younger than 18 years or by patients older than 18 years.

### DNA constructs

The SPECC1L::NTRK2 pFTRE GFP construct has been previously published by our group^5^. WT NTRK2 was PCR amplified from patient cDNA (as described above). NTRK2 ITD with flaking EcoRI and NheI restriction sites was synthesized by Twist Biosciences into the pTWIST Amp High Copy vector (see Supplementary Material for complete sequence). WT NTRK2 and NTRK2 ITD were cloned or sub-cloned by restriction digest into the pFTRE GFP lentiviral vector using EcoRI and NheI restriction sites according to standard procedures. NTRK2 ITD sequences containing tyrosine (Y) to phenylalanine (F) substitution encoding mutations (NTRK2 ITD Y516F, NTRK2 ITD Y534F, NTRK2 ITD Y658F and NTRK2 ITD Y516F/Y534F/Y658F) were synthesized by Twist Biosciences and sub-cloned into pFTRE GFP according to the procedure described above.

### Cell culture and lentiviral transfection and transduction

HEK293T and SH-SY5Y cells were maintained in DMEM (Thermo Fisher Scientific, catalogue number: 11995-065) supplemented with 10% fetal bovine serum (FBS, Thermo Fisher Scientific, catalogue number: 10100147), at 37 °C and 5% CO_2_. Ba/F3 cell lines were maintained in RPMI 1640 medium (Thermo Fisher Scientific, catalogue number: 22400-089) supplemented with 10% FBS and 0.5 ng/mL mouse interleukin-3 (IL-3) recombinant protein (PeproTech, catalogue number: 213-13-10), at 37 °C and 5% CO_2_. Lentiviral transfection of HEK293T cells and transduction of Ba/F3 and SH-SY5Y cells with NTRK2 variants was performed using Effectene Transfection Reagent (QIAGEN, catalogue number: 301425), as previously described^5^. Transduced cell lines were sorted for GFP expression by fluorescence-activated cell sorting (FACS) using the FACSAria™ Fusion (BD Biosciences).

### Western blotting

Membrane and cytosolic fractions were isolated from cell pellets using the Mem-PER™ Plus Membrane Protein Extraction Kit (Thermo Fisher Scientific, catalogue number: 89842), according to the Manufacturer’s protocol. Whole cell lysates were isolated by lysing cells in RIPA buffer according to standard procedures. Cell fractions and whole cell lysates were quantified using the Pierce™ BCA Protein Assay (Thermo Fisher Scientific, catalogue number: 23225). 10-30ug of protein lysate was separated by SDS-PAGE under denaturing conditions and proteins were transferred onto Amersham™ Protran® nitrocellulose membrane (Merck, catalogue number: GE10600016) using the Criterion™ or Mini-PROTEAN Tetra Cell systems according to the Manufacturer’s protocols (Bio-Rad Laboratories). Western blotting was performed with primary and secondary antibodies detailed in Table 1. Antibodies were detected using Immobilon Forte (Merck Millipore, catalogue number: WBLUF0500) or Clarity Max™ ECL HRP substrate reagents (Bio-Rad Laboratories, catalogue number: 1705062) and the Chemidoc™ Imaging System (Bio-Rad Laboratories). Chemiluminescent images were analyzed using Image Lab (v6.1, Bio-Rad Laboratories).

**Table 1:** Western blot antibodies

Antibody	Supplier	Catalogue number	RRID
TrkB (phospho Y705) (phospho Y706 in human)	Abcam	ab229908	AB_2892153
Anti-TrkA (phospho Y496) + TrkB (phospho Y516) + TrkC (phospho Y516) antibody	Abcam	ab197071	N/A
Pan-Trk	Abcam	ab181560	AB_2940902
Phospho-STAT5 (Tyr694)	Cell Signaling Technology	9359	AB_823649
STAT5	Cell Signaling Technology	4459	AB_10693429
Phospho-AKT (Ser473)	Cell Signaling Technology	4060	AB_2315049
AKT	Cell Signaling Technology	4691	AB_915783
Phospho-MEK1/2 (Ser217/221)	Cell Signaling Technology	9154	AB_2138017
MEK 1/2	Cell Signaling Technology	9122	AB_823567
Pan-Cadherin	Cell Signaling Technology	4068	AB_2158565
COX IV	Cell Signaling Technology	4850	AB_2085424
Bak	Sigma Aldrich	B5897	AB_258581
β-actin	Sigma Aldrich	A2228	AB_476697
Anti-rabbit HRP-conjugated	Cytiva	NA9340	AB_772191
Anti-mouse HRP-conjugated	Cytiva	NA931	AB_772210

### IL-3 withdrawal assays

IL-3 withdrawal was performed on Ba/F3 cells as previously described, with either (i) percentage viable cells, determined by PI exclusion measured by flow cytometry^7^, or (ii) number of viable cells, determined by trypan blue exclusion^5^ used as read-outs. Ba/F3 cells were cultured in media containing 0.1 ng/ml of mIL-3 for low IL-3 condition and 100 ng/ml of BDNF (PeproTech, catalogue number: 450-02-10UG) for BDNF condition. Data were analyzed using GraphPad Prism Software. Groups were compared using unpaired *t* tests with Bonferroni-Dunn correction for multiple comparisons. Significance is defined as *P value ≤0.05, **P value ≤0.01, ***P value ≤0.001.

### Cell surface staining

Live cells were washed and stained with Human TrkB Alexa Fluor® 647-conjugated Antibody (R&D Systems, RRID: AB_2892614). Unstained and stained cells were analyzed by flow cytometry using the LSRFortessa™ (BD Biosciences). Data were analyzed using FlowJo™ (FlowJo LLC).

### Drug screening

Drug screening and data analysis were performed as previously described^22^. In brief, Ba/F3 cells transformed by either SPECC1L::NTRK2 or NTRK2 ITD (maintained in the absence of IL-3) and control cells, pFTRE and NTRK2 cultured in IL-3, were seeded at 2.5 × 10^3^ cells per well in a 384 well plates, using the Multidrop Combi Reagent Dispenser (Thermo Fisher Scientific). Cells were incubated for 3 hours prior to drug addition, using the HP D300 Digital Dispenser (Tecan) and then treated with a 7-point dose titration 0.01-10,000 nM (10-fold serial dilutions) of 10 selected kinase inhibitors (MedChemExpress; see Table 2) for 72 hours. Cell viability was measured using Alamar Blue Reagent. Drug testing was performed in triplicates in three biologically independent repeat experiments (*n* = 3). Cell viability values were used to generate dose response curves using nonlinear regression analysis and determine half-maximal inhibitory values (IC50).

**Table 2:** Kinase inhibitors

Drug name	Target	Supplier	Catalogue number
Larotrectinib	Trk Receptor	MedChemExpress	HY-12866A
Selitrectinib	Trk Receptor	MedChemExpress	HY-101977
Entrectinib	ALK; ROS; Trk Receptor	MedChemExpress	HY-12678
Repotrectinib	ALK; ROS; Trk Receptor	MedChemExpress	HY-103022
Trametinib	MEK	MedChemExpress	HY-10999
Pimasertib	MEK	MedChemExpress	HY-12042
Paxalisib	mTOR; PI3K	MedChemExpress	HY-19962
Ruxolitinib	JAK	MedChemExpress	HY-50856
Dasatinib	Bcr-Abl; Src	MedChemExpress	HY-10181
Imatinib	Bcr-Abl; c-Kit; PDGFR	MedChemExpress	HY-15463

For entrectinib treatment of SH-SY5Y cells, cells were seeded at 4.2 × 10^3^ cells per well in 96-well plates and incubated for 24 hours prior to drug addition. Drug treatment was performed as above, and cells were incubated for 1 hour prior to addition of 100 ng/ml of BDNF (or vehicle). Cell viability was determined using Alamar Blue Reagent after 72 hours and drug testing was performed in triplicates in two independent experiments in two biologically independent cell lines (*n* = 4). Cell viability values were used to generate dose response curves using nonlinear regression analysis and determine half-maximal inhibitory values (IC50). Mean IC50 values were compared between BDNF treated and standard conditions using Extra sum-of-squares F Test. Data were analyzed using GraphPad Prism Software (version 10.2.3, GraphPad Prism Software, LLC).

### In silico protein structure analysis

ESMFold (esmfold_v1) was used to predict the structural conformation of NTRK2 ITD and WT NTRK2 proteins^11^. US-align^12^ was used to superimpose NTRK2 ITD and WT NTRK2 proteins. The resulting aligned PDB files were visualized and colored using ChimeraX-1.6.1.

## Supplementary information

Supplementary Information

## Data Availability

All data generated or analysed during this study are included in this published article and its supplementary information files. Raw data files are available upon request from the corresponding author. RNAseq and WGS data were generated as part of the ZERO program and are available upon request to https://www.zerochildhoodcancer.org.au/clinicians-researchers/for-researchers/data-and-sample-resources.

## References

[1] Wong, M. et al. Whole genome, transcriptome and methylome profiling enhances actionable target discovery in high-risk pediatric cancer. *Nature. Med*. **26**, 1742–1753 (2020). 33020650 10.1038/s41591-020-1072-4

[2] Lau, L. M. S. et al. Precision-guided treatment in high-risk pediatric cancers. *Nature. Med.* **30**, 1913–1922 (2024). 38844796 10.1038/s41591-024-03044-0 PMC11271405

[3] Slayton, W. B. et al. Dasatinib Plus Intensive Chemotherapy in Children, Adolescents, and Young Adults With Philadelphia Chromosome-Positive Acute Lymphoblastic Leukemia: Results of Children’s Oncology Group Trial AALL0622. *J. Clin. Oncol.* **36**, 2306 (2018). 29812996 10.1200/JCO.2017.76.7228 PMC6067800

[4] Laetsch, T. W. et al. Larotrectinib for paediatric solid tumours harbouring NTRK gene fusions: phase 1 results from a multicentre, open-label, phase 1/2 study. *Lancet Oncol.* **19**, 705–714 (2018). 29606586 10.1016/S1470-2045(18)30119-0 PMC5949072

[5] Khuong-Quang, D. A. et al. Recurrent SPECC1L-NTRK fusions in paediatric sarcoma and brain tumours.*Cold Spring Harb. Mol. Case Stud.* **6**, a05710 (2020). 10.1101/mcs.a005710 PMC7784491 33144287

[6] Brown, L. M. et al. Targeted therapy and disease monitoring in CNTRL-FGFR1-driven leukaemia. *Pediatr. blood cancer* **66**, e27897 (2019). 31250523 10.1002/pbc.27897

[7] Brown, L. M. et al. SFPQ-ABL1 and BCR-ABL1 use different signaling networks to drive B-cell acute lymphoblastic leukemia. *Blood Adv.* **6**, 2373–2387 (2022). 35061886 10.1182/bloodadvances.2021006076 PMC9006296

[8] Sadras, T. et al. Unusual PDGFRB fusion reveals novel mechanism of kinase activation in Ph-like B-ALL. *Leukemia*. **37**, 905–909 (2023). 36810896 10.1038/s41375-023-01843-x PMC10079538

[9] Iyer, R. et al. Entrectinib is a potent inhibitor of Trk-driven neuroblastomas in a xenograft mouse model. *Cancer Lett.* **372**, 179–186 (2016). 26797418 10.1016/j.canlet.2016.01.018 PMC4792275

[10] Meakin, S. O., MacDonald, J. I. S., Gryz, E. A., Kubu, C. J. & Verdi, J. M. The signaling adapter FRS-2 competes with Shc for binding to the nerve growth factor receptor TrkA. A model for discriminating proliferation and differentiation. *J. Biol. Chem.* **274**, 9861–9870 (1999). 10092678 10.1074/jbc.274.14.9861

[11] Lin, Z. et al. Evolutionary-scale prediction of atomic-level protein structure with a language model. *Science* **379**, 1123–1130 (2023). 36927031 10.1126/science.ade2574

[12] Zhang, C., Shine, M., Pyle, A. M. & Zhang, Y. US-align: universal structure alignments of proteins, nucleic acids, and macromolecular complexes. *Nat. Methods* **19**, 1109–1115 (2022). 36038728 10.1038/s41592-022-01585-1

[13] Rücker, F. G. et al. Molecular landscape and prognostic impact of FLT3-ITD insertion site in acute myeloid leukemia: RATIFY study results. *Leukemia* **36**, 90–99 (2022). 34316017 10.1038/s41375-021-01323-0 PMC8727286

[14] Ryall, S. et al. Integrated Molecular and Clinical Analysis of 1,000 Pediatric Low-Grade Gliomas. *Cancer Cell* **37**, 569–583.e565 (2020). 32289278 10.1016/j.ccell.2020.03.011 PMC7169997

[15] Bagger, F. O. et al. Whole genome sequencing in clinical practice. *BMC Med. Genomics* **17**, 39 (2024). 38287327 10.1186/s12920-024-01795-w PMC10823711

[16] Langenberg, K. P. S., Looze, E. J. & Molenaar, J. J. The Landscape of Pediatric Precision Oncology: Program Design, Actionable Alterations, and Clinical Trial Development. *Cancers (Basel)* **13**, 4324 (2021). 34503139 10.3390/cancers13174324 PMC8431194

[17] Drilon, A. et al. Efficacy of Larotrectinib in TRK Fusion-Positive Cancers in Adults and Children. *N. Engl. J. Med.* **378**, 731–739 (2018). 29466156 10.1056/NEJMoa1714448 PMC5857389

[18] Korshunov, A. et al. Molecular analysis of pediatric CNS-PNET revealed nosologic heterogeneity and potent diagnostic markers for CNS neuroblastoma with FOXR2-activation. *Acta Neuropathol. Commun.* **9**, 20 (2021). 33536079 10.1186/s40478-021-01118-5 PMC7860633

[19] Cocco, E., Scaltriti, M. & Drilon, A. NTRK fusion-positive cancers and TRK inhibitor therapy. *Nat. Rev. Clin. Oncol.* **15**, 731–747 (2018). 30333516 10.1038/s41571-018-0113-0 PMC6419506

[20] Janoschke, M. et al. Efficient integration of transmembrane domains depends on the folding properties of the upstream sequences. *Proc. Natl Acad. Sci.* **118**, e2102675118 (2021). 34373330 10.1073/pnas.2102675118 PMC8379923

[21] Ojemalm, K., Halling, K. K., Nilsson, I. & von Heijne, G. Orientational preferences of neighboring helices can drive ER insertion of a marginally hydrophobic transmembrane helix. *Mol. Cell* **45**, 529–540 (2012). 22281052 10.1016/j.molcel.2011.12.024 PMC3553544

[22] Mayoh, C. et al. High-Throughput Drug Screening of Primary Tumor Cells Identifies Therapeutic Strategies for Treating Children with High-Risk Cancer. *Cancer Res* **83**, 2716–2732 (2023). 37523146 10.1158/0008-5472.CAN-22-3702 PMC10425737

[23] Zhou, X. et al. Exploring genomic alteration in pediatric cancer using ProteinPaint. *Nat. Genet.* **48**, 4–6 (2016). 26711108 10.1038/ng.3466 PMC4892362

