# Supplementary material for: A novel TRKB-activating internal tandem duplication characterizes a new mechanism of receptor tyrosine kinase activation
Source: NPJ Precis Oncol. 2025 May 10;9:137. doi: 10.1038/s41698-025-00928-3 (PMC12065843; doi:10.1038/s41698-025-00928-3)
Supplement: Supplementary file 1 — Supplementary Information [file 41698_2025_928_MOESM1_ESM.pdf]

A novel TRKB-activating internal tandem duplication characterizes a new mechanism of receptor tyrosine kinase activation

Lauren M. Brown[1, 2], Gabor Tax[1, 2], Pablo Acera Mateos[1, 2], Antoine de Weck[1, 2], Steve Foresto[3], Thomas Robertson[3], Fatimah Jalud[4, 5], Pamela Ajuyah[1], Paulette Barahona[1], Jie Mao[1], M Emmy M. Dolman[1, 2], Marie Wong[1, 2], Chelsea Mayoh[1, 2], Mark J. Cowley[1, 2], Loretta M.S. Lau[1, 2, 6], Teresa Sadras[4, 5], and Paul G. Ekert[1, 2, 4, 5, 7]

Supplementary Material

NTRK2 ITD sequence

```
>EcoRI-NTRK2-ITD-NheI
GAATTCATGTCGTCCTGGATAAGGTGGCATGGACCCGCCATGGCGCGGCTCTGGGGCTTCTGCTGGCTGGTTGTGGG
CTTCTGGAGGGCCGCTTTCGCCTGTCCCACGTCCTGCAAATGCAGTGCCTCTCGGATCTGGTGCAGCGACCCTTCTC
CTGGCATCGTGGCATTTCCGAGATTGGAGCCTAACAGTGTAGATCCTGAGAACATCACCGAAATTTTCATCGCAAAC
CAGAAAAGGTTAGAAATCATCAACGAAGATGATGTTGAAGCTTATGTGGGACTGAGAAATCTGACAATTGTGGATTC
TGGATTAAAATTTGTGGCTCATAAAGCATTTCTGAAAAACAGCAACCTGCAGCACATCAATTTTACCCGAAACAAAC
TGACGAGTTTGTCTAGGAAACATTTCCGTCACCTTGACTTGTCTGAACTGATCCTGGTGGGCAATCCATTTACATGC
TCCTGTGACATTATGTGGATCAAGACTCTCCAAGAGGCTAAATCCAGTCCAGACACTCAGGATTTGTACTGCCTGAA
TGAAAGCAGCAAGAATATTCCCCTGGCAAACCTGCAGATACCCAATTGTGGTTTGCCATCTGCAAATCTGGCCGCAC
CTAACCTCACTGTGGAGGAAGGAAAGTCTATCACATTATCCTGTAGTGTGGCAGGTGATCCGGTTCCTAATATGTAT
TGGGATGTTGGTAACCTGGTTTCCAAACATATGAATGAAACAAGCCACACACAGGGCTCCTTAAGGATAACTAACAT
TTCATCCGATGACAGTGGGAAGCAGATCTCTTGTGTGGCGGAAAATCTTGTAGGAGAAGATCAAGATTCTGTCAACC
TCACTGTGCATTTTGCACCAACTATCACATTTCTCGAATCTCCAACCTCAGACCACCACTGGTGCATTCCATTCACT
GTGAAAGGCAACCCCAAACCAGCGCTTCAGTGGTTCTATAACGGGGCAATATTGAATGAGTCCAAATACATCTGTAC
TAAAATACATGTTACCAATCACACGGAGTACCACGGCTGCCTCCAGCTGGATAATCCCACTCACATGAACAATGGGG
ACTACACTCTAATAGCCAAGAATGAGTATGGGAAGGATGAGAAACAGATTTCTGCTCACTTCATGGGCTGGCCTGGA
ATTGACGATGGTGCAAACCCAAATTATCCTGATGTAATTTATGAAGATTATGGAACTGCAGCGAATGACATCGGGGA
CACCACGAACAGAAGTAATGAAATCCCTTCCACAGACGTCACTGATAAAACCGGTCGGGAACATCTCTCGGTCTATG
CTGTGGTGGTGATTGCGTCTGTGGTGGGATTTTGCCTTTTGGTAATGCTGTTTCTGCTTAAGTTGGCAAGACACTCC
AAGTTTGGCATGAAAGGCCCAGCCTCCGTTATCAGCAATGATGATGACTCTGCCAGCCCACTCCATCACATCTCCAA
TGGGAGTAACACTCCATCTTCTTCGGAAGGTGGCCCAGATGCTGTCATTATTGGAATGACCAAGATCCCTGTCATTG
AAAATCCCCAGTACTTTGGCATCACCAACAGTCAGCTCAAGCCAGACACATGTGCAAACCCAAATTATCCTGATGTA
ATTTATGAAGATTATGGAACTGCAGCGAATGACATCGGGGACACCACGAACAGAAGTAATGAAATCCCTTCCACAGA
CGTCACTGATAAAACCGGTCGGGAACATCTCTCGGTCTATGCTGTGGTGGTGATTGCGTCTGTGGTGGGATTTTGCC
TTTTGGTAATGCTGTTTCTGCTTAAGTTGGCAAGACACTCCAAGTTTGGCATGAAAGGCCCAGCCTCCGTTATCAGC
AATGATGATGACTCTGCCAGCCCACTCCATCACATCTCCAATGGGAGTAACACTCCATCTTCTTCGGAAGGTGGCCC
AGATGCTGTCATTATTGGAATGACCAAGATCCCTGTCATTGAAAATCCCCAGTACTTTGGCATCACCAACAGTCAGC
TCAAGCCAGACACATTTGTTCAGCACATCAAGCGACATAACATTGTTCTGAAAAGGGAGCTAGGCGAAGGAGCCTTT
GGAAAAGTGTTCCTAGCTGAATGCTATAACCTCTGTCCTGAGCAGGACAAGATCTTGGTGGCAGTGAAGACCCTGAA
GGATGCCAGTGACAATGCACGCAAGGACTTCCACCGTGAGGCCGAGCTCCTGACCAACCTCCAGCATGAGCACATCG
TCAAGTTCTATGGCGTCTGCGTGGAGGGCGACCCCCTCATCATGGTCTTTGAGTACATGAAGCATGGGGACCTCAAC
```

AAGTTCCTCAGGGCACACGGCCCTGATGCCGTGCTGATGGCTGAGGGCAACCCGCCCACGGAACTGACGCAGTCGCA
GATGCTGCATATAGCCCAGCAGATCGCCGCGGGCATGGTCTACCTGGCGTCCCAGCACTTCGTGCACCGCGATTTGG
CCACCAGGAACTGCCTGGTCGGGGAGAACTTGCTGGTGAAAATCGGGGACTTTGGGATGTCCCGGGACGTGTACAGC
ACTGACTACTACAGGGTCGGTGGCCACACAATGCTGCCCATTCGCTGGATGCCTCCAGAGAGCATCATGTACAGGAA
ATTCACGACGGAAAGCGACGTCTGGAGCCTGGGGGTCGTGTTGTGGGAGATTTTCACCTATGGCAAACAGCCCTGGT
ACCAGCTGTCAAACAATGAGGTGATAGAGTGTATCACTCAGGGCCGAGTCCTGCAGCGACCCCGCACGTGCCCCCAG
GAGGTGTATGAGCTGATGCTGGGGTGCTGGCAGCGAGAGCCCCACATGAGGAAGAACATCAAGGGCATCCATACCCT
CCTTCAGAACTTGGCCAAGGCATCTCCGGTCTACCTGGACATTCTAGGCTAGGCTAGC

Supplementary Tables

Supplementary Table 1. IC50 values from kinase inhibitor drug screen in Ba/F3 cells

	**pFTRE + IL-3**	**NTRK2 + IL-3**	**SPECC1L::NTRK2**	**NTRK2 ITD**
	Mean IC50 (nM) [95% CI]			
**Larotrectinib**	10625 [*undefined*]	*Unstable*	11.8 [11.3, *undefined*]	30.6 [25.8, 35.7]
**Selitrectinib**	71912 [*undefined*]	46994 [*undefined*]	1.7 [1.4, 2.0]	3.4 [3.0, 3.8]
**Entrectinib**	782 [622, 896]	1523 [1421, 5834]	5.9 [4.0, 7.1]	10.1 [9.7, 10.7]
**Repotrectinib**	169 [145, 194]	315 [268, 365]	0.83 [0.71, *undefined*]	1.5 [*undefined*, 1.9]
**Trametinib**	28.2 [14.9, 62.0]	105.1 [44.8, 378]	2028 [282, *undefined*]	12.3 [8.7, 18.3]
**Pimasertib**	183 [84.1, 749]	431 [168, 6290]	6350 [*undefined*]	107 [86.6, 137]
**Paxalisib**	16536 [3181, *undefined*]	2965 [1630, 12436]	574.1 [423, 774]	184 [154, 222]
**Ruxolitinib**	61.8 [47.8, 79.0]	158 [117, 219]	2079 [1780, *undefined*]	1661 [1108, 5780]
**Dasatinib**	*Unstable*	1721 [1445, *undefined*]	*Unstable*	*Unstable*
**Imatinib**	1296 [*undefined*]	1680 [*undefined*]	*Unstable*	*Unstable*

Supplementary Figures

```
>NTRK2 ITD protein
MSSWIRWHGPAMARLWGFCWLVVGFWRAAFACPTSCKCSASRIWCSDPSPGIVAFPRLEPNSVDPENITEIFIANQK
RLEIINEDDVEAYVGLRNLTIVDSGLKFVAHKAFLKNSNLQHINFTRNKLTSLSRKHFRHLDLSELILVGNPFTCSC
DIMWIKTLQEAKSSPDTQDLYCLNESSKNIPLANLQIPNCGLPSANLAAPNLTVEEGKSITLSCSVAGDPVPNMYWD
VGNLVSKHMNETSHTQGSLRITNISSDDSGKQISCVAENLVGEDQDSVNLTVHFAPTITFLESPTSDHHWCIPFTVK
GNPKPALQWFYNGAILNESKYICTKIHVTNHTEYHGCLQLDNPTHMNNGDYTLIAKNEYGKDEKQISAHFMGWPGID
DGANPNYPDVIYEDYGTAANDIGDTTNRSNEIPSTDVTDKTGREHLSVYAVVVIASVVGFCLLVMLFLLKLARHSKF
GMKGPASVISNDDDSASPLHHISNGSNTPSSSEGGPDAVIIGMTKIPVIENPQYFGITNSQLKPDTCANPNYPDVIY
EDYGTAANDIGDTTNRSNEIPSTDVTDKTGREHLSVYAVVVIASVVGFCLLVMLFLLKLARHSKFGMKGPASVISND
DDSASPLHHISNGSNTPSSSEGGPDAVIIGMTKIPVIENPQYFGITNSQLKPDTFVQHIKRHNIVLKRELGEGAFGK
VFLAECYNLCPEQDKILVAVKTLKDASDNARKDFHREAELLTNLQHEHIVKFYGVCVEGDPLIMVFEYMKHGDLNKF
LRAHGPDAVLMAEGNPPTELTQSQMLHIAQQIAAGMVYLASQHFVHRDLATRNCLVGENLLVKIGDFGMSRDVYSTD
YYRVGGHTMLPIRWMPPESIMYRKFTTESDVWSLGVVLWEIFTYGKQPWYQLSNNEVIECITQGRVLQRPRTCPQEV
YELMLGCWQREPHMRKNIKGIHTLLQNLAKASPVYLDILG
```

KEY

C additional residue (Cys387) as a result of ITD

DUPLICATION (Ala388-Thr528 of WT NTRK2)

Supplementary Figure 1. NTRK2 ITD protein sequence

NTRK2 ITD protein sequence with duplicated region colored according to the key. Duplication of exons 10-13 results in the insertion of an additional 142 amino acids, 141 of which are homologous to Ala388-Thr528 of WT NTRK2. The additional Cystine residue is due to the codon being split across the exon 10 boundary.

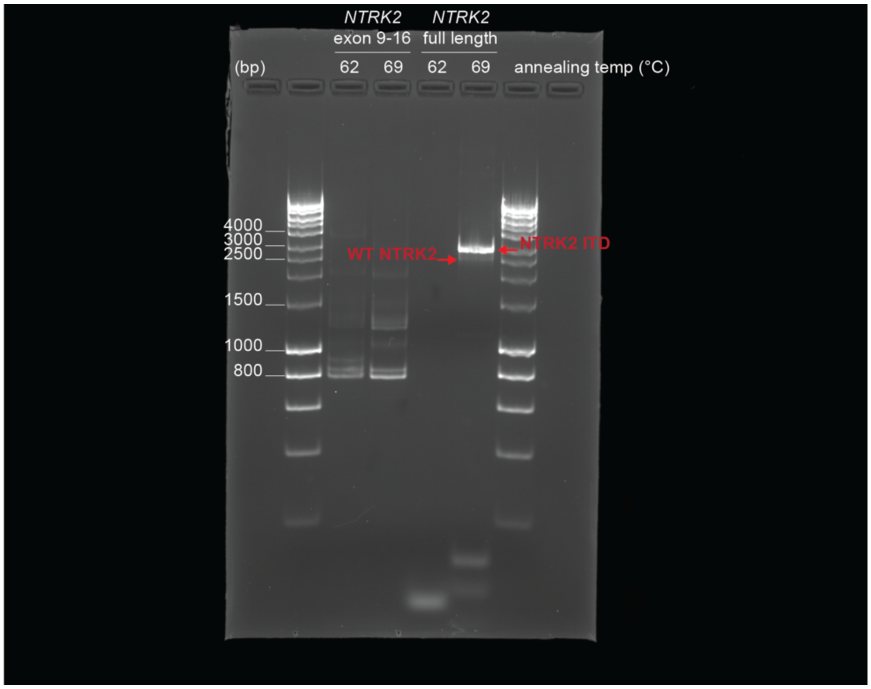

Supplementary Figure 2. Uncropped DNA gel image of PCR amplification of NTRK2 ITD full length and duplicated region from a CNS neuroblastoma patient

PCR amplification of NTRK2 ITD duplicated region or full-length product using primers targeting exon 9 and exon 16 of *NTRK2* (exon 9-16) or 5' and 3' ends of *NTRK2* (full length) using either 62°C or 69°C. PCR amplification was predicted to yield bands at 824bp for wild type *NTRK2* or 1,436bp for *NTRK2* ITD using exon 9-16 primers or 2,533bp for wild type or 3,007bp for ITD. PCR amplification with exon 9-16 primers produced multiple non-specific products but amplification of full-length NTRK2 ITD and WT NTRK2 was successful using an annealing temperature of 69°C and are indicated by the red arrows.

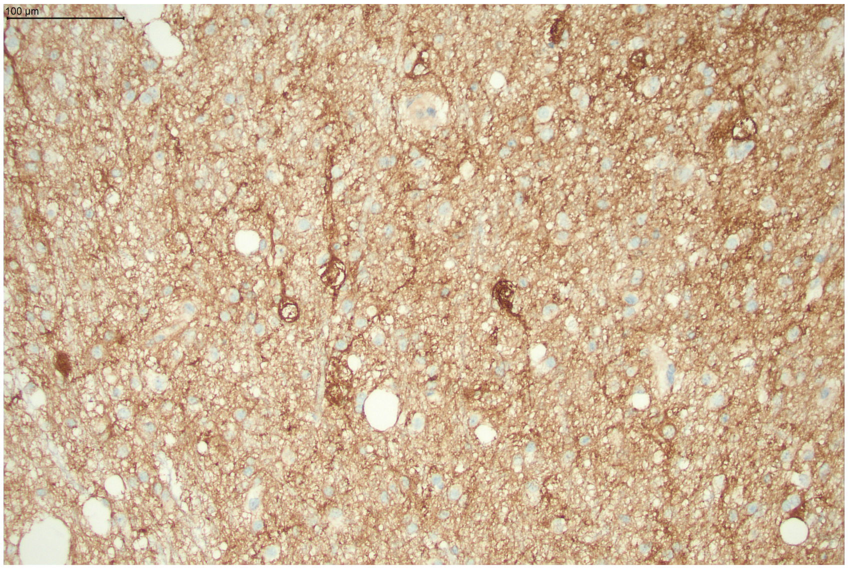

Supplementary Figure 3. Immunohistochemistry analysis of pan-TRK expression in normal brain tissue

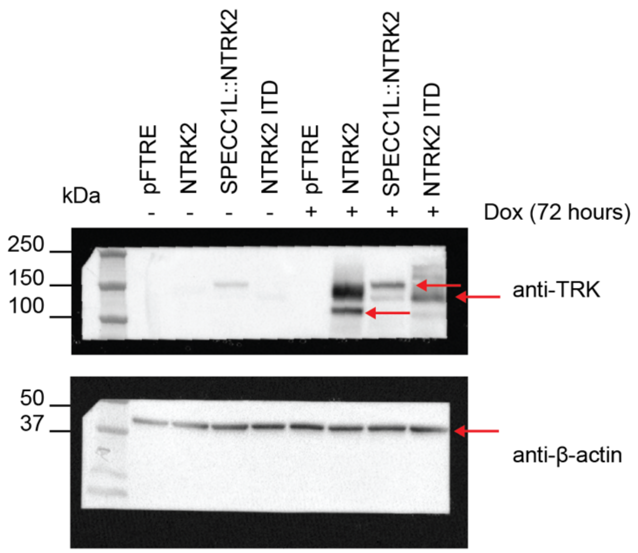

Supplementary Figure 4. Uncropped original Western blots of doxycycline-induced expression of NTRK2 variants in Ba/F3 cells

Western blot analysis of TRK and β-actin (loading control) expression in Ba/F3 cells treated with doxycycline for 72 expression to induce expression of NTRK2, SPECC1L::NTRK2 or NTRK2 ITD (Shown in Figure 2a). pFTRE represents the empty vector control. The locations of molecular weight markers are indicated (kDa). The location of the target protein is indicated by the red arrows. Chemiluminescent images are merged with colorimetric images that were sequentially captured to determine target protein size against the molecular weight marker, using Image Lab 6.1 software (Bio-Rad Laboratories).

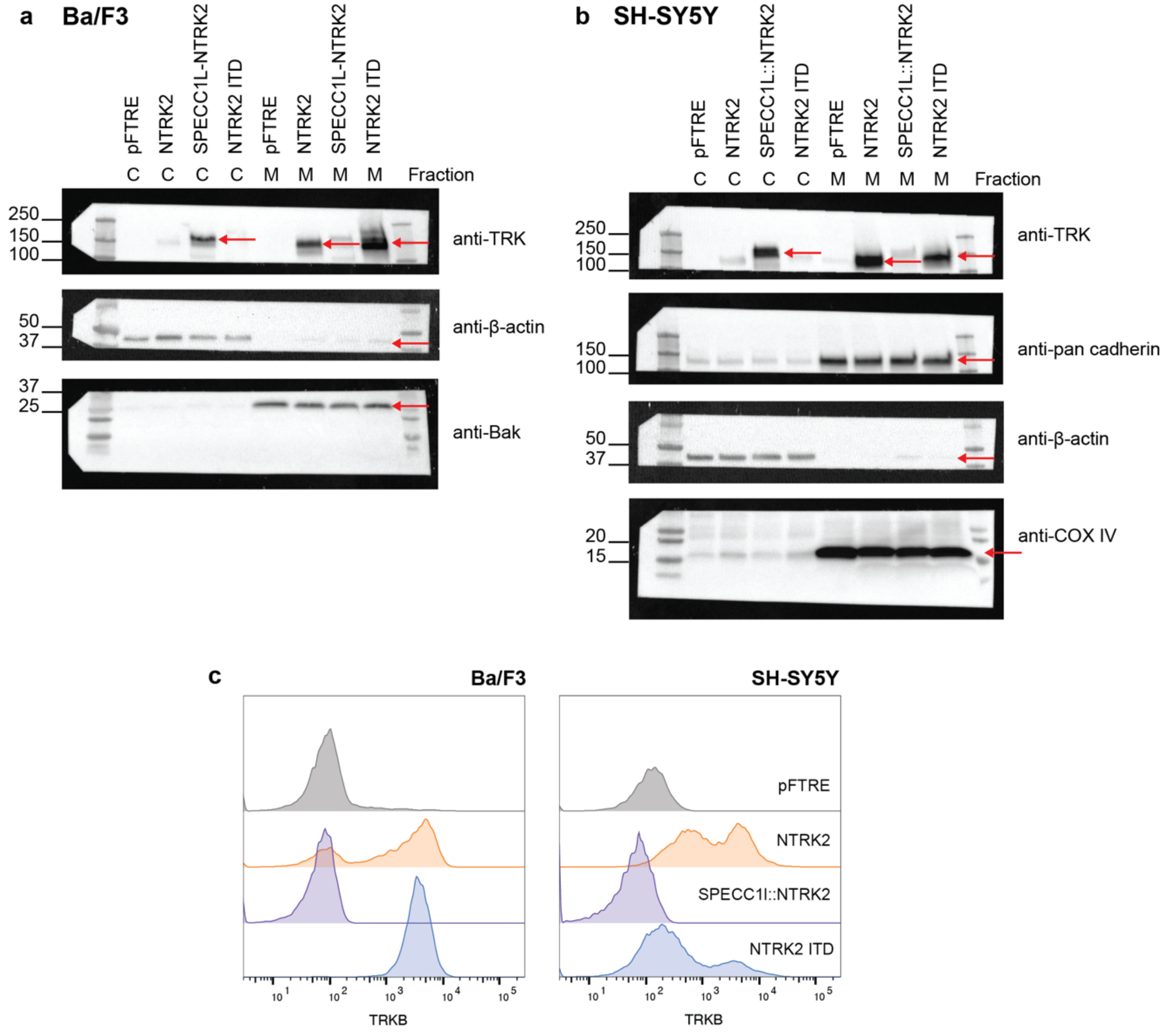

Supplementary Figure 5. Analysis of NTRK2 ITD localization in Ba/F3 and SH-SY5Y cells

a-b) Western blot analysis of cytoplasmic (C) and membrane (M) protein fractions derived from Ba/F3 (a) or SH-SY5Y (b, shown in Figure 2b) cells with dox-induced expression of NTRK2 variants. pFTRE represents the empty vector control. For Ba/F3, β-actin and bak were used as cytoplasmic and membrane markers, respectively. For SH-SY5Y, β-actin, pan-cadherin, and COX IV were used as cytoplasmic, plasma membrane, and mitochondrial membrane markers, respectively. The locations of molecular weight markers are indicated (kDa). The location of the target protein is indicated by the red arrows, where SPECC1L::NTRK2 is predominantly

expressed in the cytoplasm, and NTRK2 and NTRK2 ITD in the membrane in both cell lines. Chemiluminescent images are merged with colorimetric images that were sequentially captured to determine target protein size against the molecular weight marker, using Image Lab 6.1 software (Bio-Rad Laboratories). c) Cell surface expression of TRKB in Ba/F3 and SH-SY5Y cells transduced with NTRK2 variants, determined by cell surface staining with an anti-TRK antibody and flow cytometry analysis. These images represent the second biological repeat.

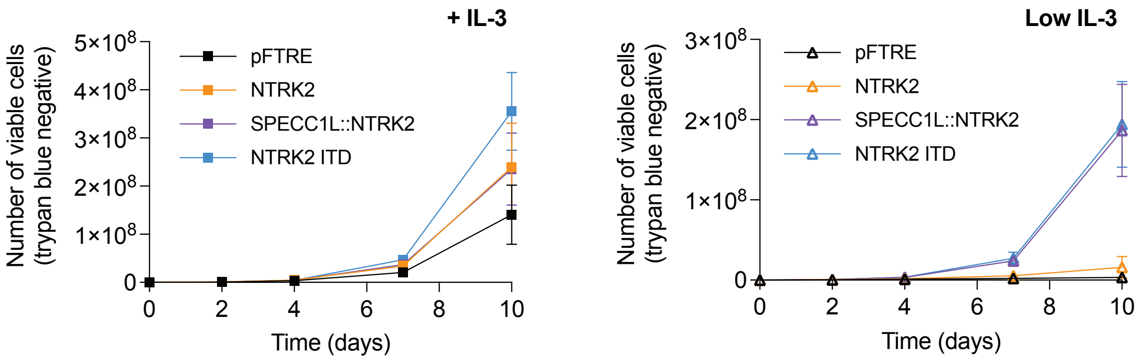

Supplementary Figure 6. +IL-3 and low IL-3 conditions for IL-3 withdrawal assay performed in Ba/F3 cells expressing NTRK2 ITD

Graphs show number of viable (determined by trypan blue exclusion) Ba/F3 cells cultured in the presence of IL-3 (normal conditions; +IL-3) or reduced IL-3 conditions (0.1ng/ml IL-3; low IL-3) 10-day period. Data is presented as mean ± SEM (n=3).

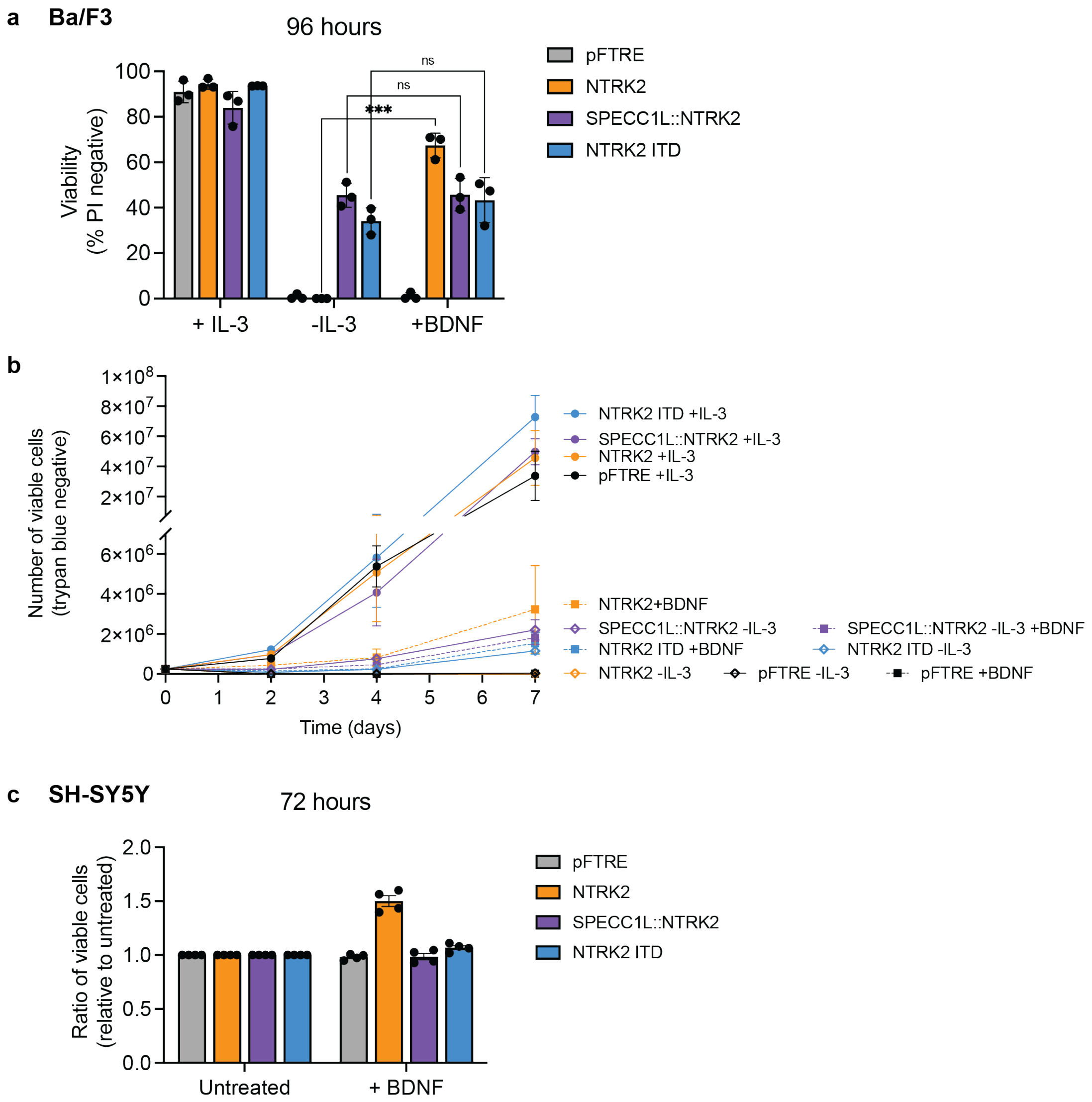

Supplementary Figure 7. BDNF does not accelerate proliferation or transformation by NTRK2 ITD

a) Viability analysis of Ba/F3 cells cultured in normal conditions (+IL-3), no IL-3 (-IL-3) or with 100ng/ml BDNF (+BDNF) for 96 hours. Viability was determined by PI exclusion measured by flow cytometry. Data is presented as mean ± SEM (n=3). Viability was compared between -IL-3 and +BDNF condition for each NTRK2 variant cell line using unpaired t tests with Bonferroni-Dunn correction for multiple comparisons (ns = not significant, *** = P value ≤0.001). b)

Number of viable (determined by trypan blue exclusion) Ba/F3 cells cultured in each condition over a 7-day period. Data is presented as mean ± SEM (n=3). c) Ratio of viable SH-SY5Y cells expressing NTRK2 variants with BDNF compared to untreated, determined using Alamar Blue assay. Mean fluorescence values of each condition were normalised to untreated cells. Data is presented as mean ± SEM (n=4).

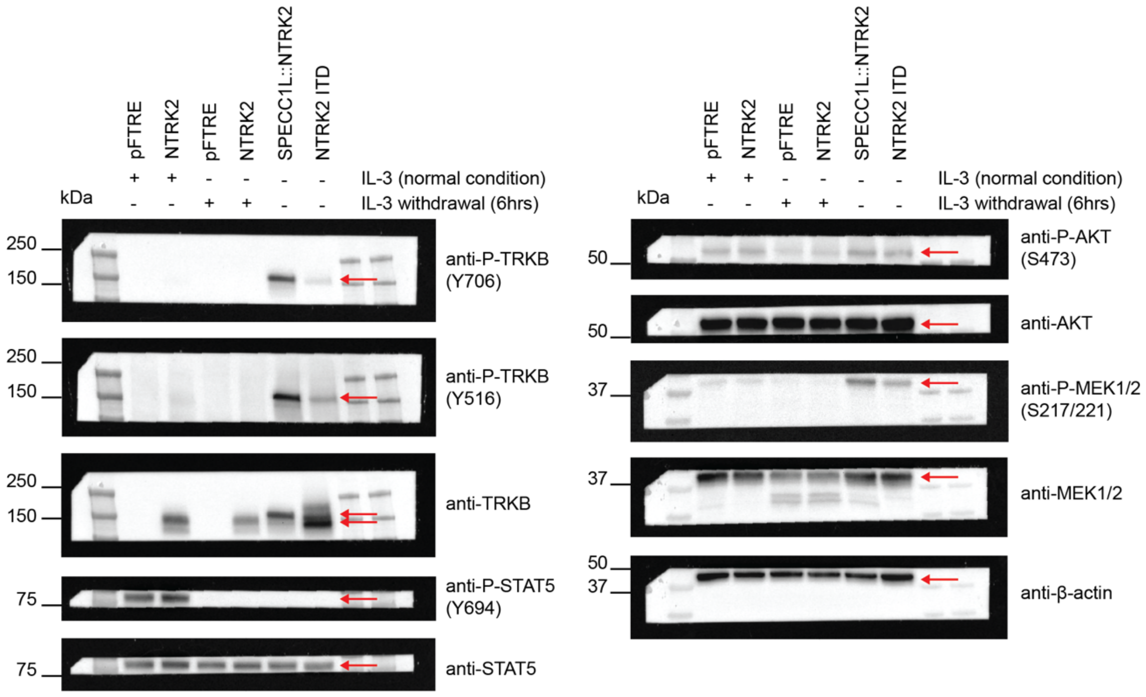

Supplementary Figure 8. Uncropped original Western blots of Ba/F3 cells transformed by SPECC1L::NTRK2 and NTRK2 ITD

Western blot analysis of TRK and downstream signaling activation in Ba/F3 cells transduced with NTRK2, SPECC1L::NTRK2, NTRK2 ITD, or empty vector control (pFTRE) (Shown in Figure 4f). Analysis was performed on pFTRE and NTRK2 Ba/F3 cells in either the presence of IL-3 or following 6-hour IL-3 withdrawal and transformed SPECC1L::NTRK2 and NTRK2 ITD Ba/F3 cells (no IL-3). The locations of molecular weight markers are indicated (kDa). The location of the target protein is indicated by the red arrows. Chemiluminescent images are merged with colorimetric images that were sequentially captured to determine target protein size against the molecular weight marker, using Image Lab 6.1 software (Bio-Rad Laboratories).

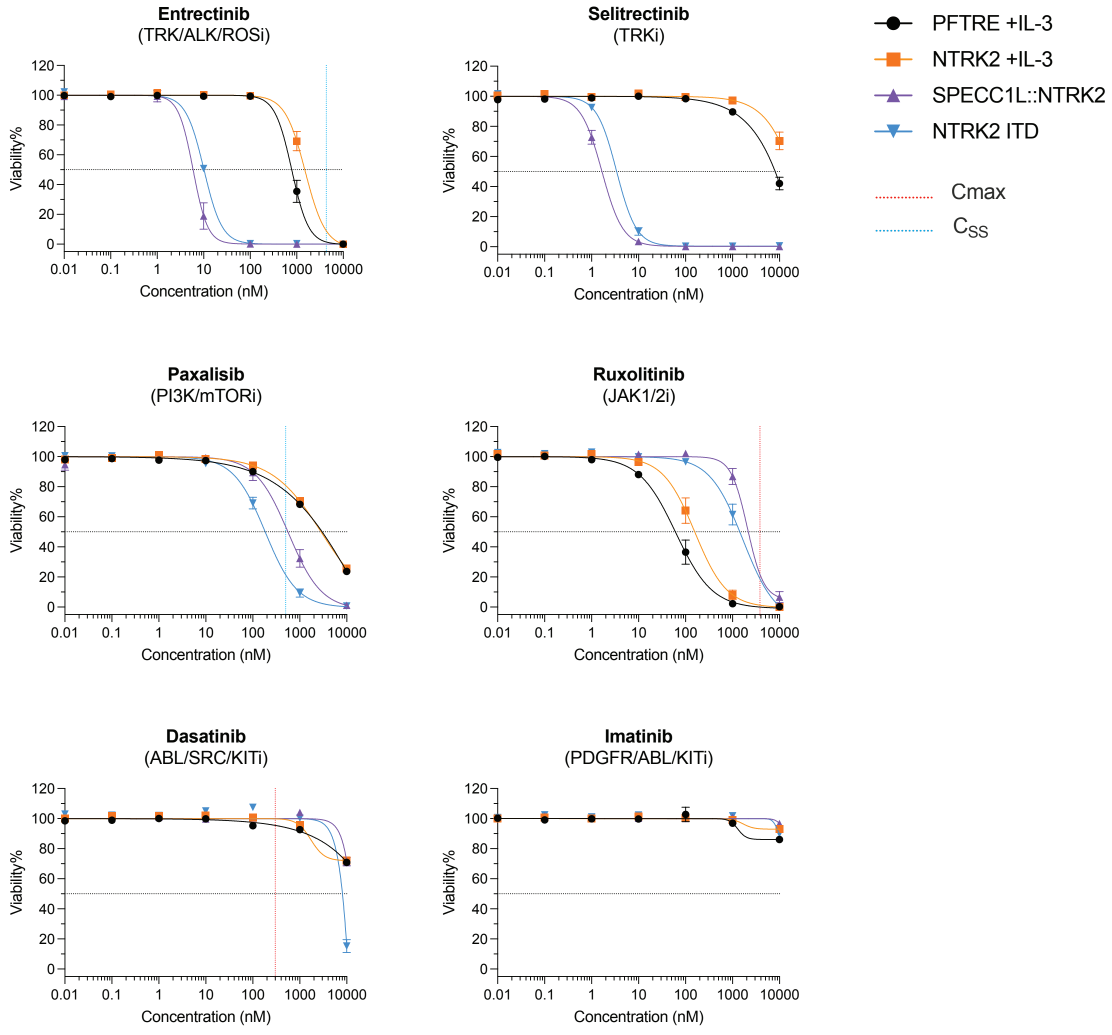

Supplementary Figure 9. Dose response curves of Ba/F3 cells expressing NTRK2 ITD treated with kinase inhibitors

Viability analysis of Ba/F3 cells treated with kinase inhibitor drugs: entrectinib, selitrectinib, paxalisib, ruxolitinib, dasatinib and imatinib. Maximum serum concentration (Cmax) and steady-state concentration (C_{ss}) of each of the drugs, if known, is represented by the red and blue dotted lines, respectively. IC_{50} values are depicted by the black dotted line. Cells were screened in technical triplicates and data is presented as mean ± SEM (n=3).

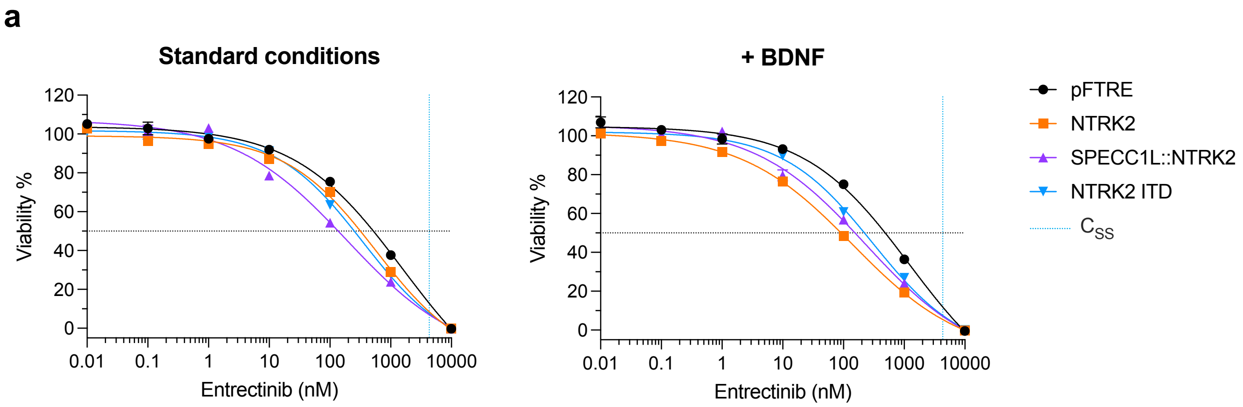

b

	Standard conditions	+BDNF (100ng/ml)	
	Mean IC50 (nM) [95% CI]		***P-value***
**pFTRE**	1572 [757, 7881]	1210 [604, 5798]	0.6981
**NTRK2**	571 [379, 1122]	141 [99, 225]	0.0003*
**SPECC1L::NTRK2**	191 [103, 515]	235 [143, 486]	0.6442
**NTRK2 ITD**	353 [248, 562]	308 [220, 473]	0.2670

Supplementary Figure 10. Dose response curves SH-SY5Y cells expressing NTRK2 variants treated with entrectinib

a) Viability analysis of SH-SY5Y cells treated with entrectinib. Steady-state concentration (C_{ss}) of entrectinib is represented by the blue dotted line. IC_{50} values are depicted by the black dotted line. Cells were screened in technical triplicates and data is presented as mean ± SEM (n=4). b) Table of entrectinib IC50 values (nM) determined by non-linear regression analysis. IC50 values were compared between cells treated under standard conditions or in the presence of BDNF using Extra sum-of-squares F Test.

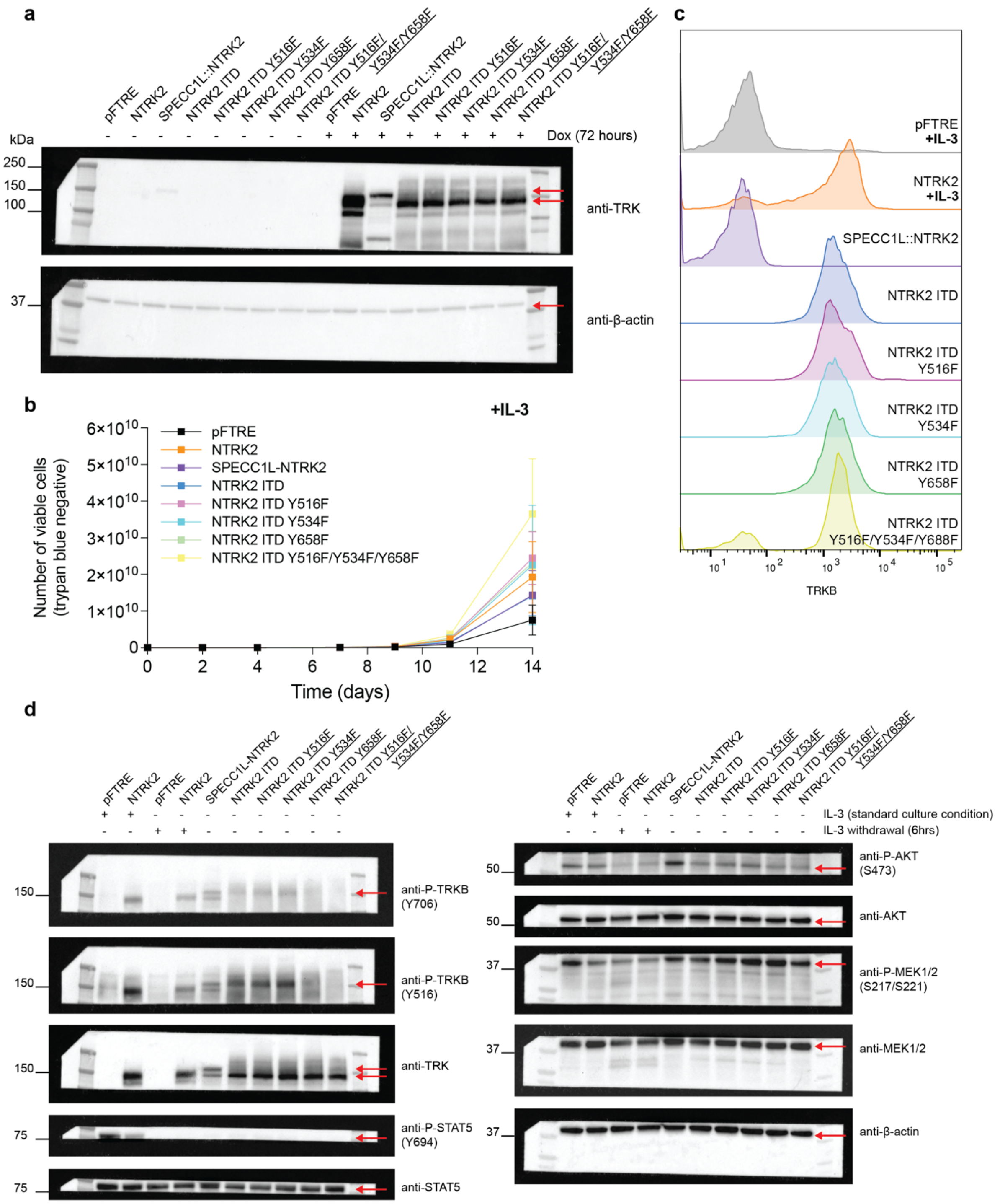
a
pFTRE
NTRK2
SPECC1L::NTRK2
NTRK2 ITD
NTRK2 ITD Y516F
NTRK2 ITD Y534F
NTRK2 ITD Y658F
NTRK2 ITD Y516F/Y534F/Y658F
Dox (72 hours)
kDa
250
150
100
anti-TRK
37
anti-β-actin
b
+IL-3
pFTRE
NTRK2
SPECC1L-NTRK2
NTRK2 ITD
NTRK2 ITD Y516F
NTRK2 ITD Y534F
NTRK2 ITD Y658F
NTRK2 ITD Y516F/Y534F/Y658F
Number of viable cells (trypan blue negative)
Time (days)
c
pFTRE +IL-3
NTRK2 +IL-3
SPECC1L::NTRK2
NTRK2 ITD
NTRK2 ITD Y516F
NTRK2 ITD Y534F
NTRK2 ITD Y658F
NTRK2 ITD Y516F/Y534F/Y688F
TRKB
d
IL-3 (standard culture condition)
IL-3 withdrawal (6hrs)
anti-P-TRKB (Y706)
anti-P-TRKB (Y516)
anti-TRK
anti-P-STAT5 (Y694)
anti-STAT5
anti-P-AKT (S473)
anti-AKT
anti-P-MEK1/2 (S217/S221)
anti-MEK1/2
anti-β-actin

Supplementary Figure 11. Generation of NTRK2 ITD Y>F mutant cell lines and functional analysis

a) Western blot of analysis of Ba/F3 cells with doxycycline (dox) inducible expression of NTRK2, SPECC1L::NTRK2, NTRK2 ITD, NTRK2 ITD Y516F, NTRK2 ITD Y534F, NTRK2 ITD Y658F, NTRK2 ITD Y516F/Y534F/Y658F, or empty vector control (pFTRE). The locations of molecular weight markers (kDa) are indicated on the left and location of the target protein is indicated by the red arrows. b) Number of viable (determined by trypan blue exclusion) Ba/F3 cells expressing NTRK2 ITD variants under normal conditions (+IL-3) over a 14-day period. Data is presented as mean ± SEM (n=3). c) Cell surface expression of TRKB in transformed NTRK2 ITD mutant Ba/F3 cells, determined by cell surface staining with an anti-Trk antibody and flow cytometry analysis. This is a representative image of two biologically independent experiments where the NTRK2 ITD triple mutant was transforming (n=2). d) Uncropped original Western blots of analysis of TRK and downstream signaling activation in Ba/F3 cells transduced with NTRK2, SPECC1L::NTRK2, NTRK2 ITD, NTRK2 variants (Y516F, Y534F, Y658F, or Y516F/Y534F/Y658F) or empty vector control (pFTRE) (Shown in Figure 4c). Analysis was performed on pFTRE and NTRK2 Ba/F3 cells in either the presence of IL-3 or following 6-hour IL-3 withdrawal and transformed SPECC1L::NTRK2 and NTRK2 ITD variants Ba/F3 cells (no IL-3). The locations of molecular weight markers (kDa) are indicated on the left and location of the target protein is indicated by the red arrows. For Western blot images, chemiluminescent images are merged with colorimetric images that were sequentially captured to determine target protein size against the molecular weight marker, using Image Lab 6.1 software (Bio-Rad Laboratories).

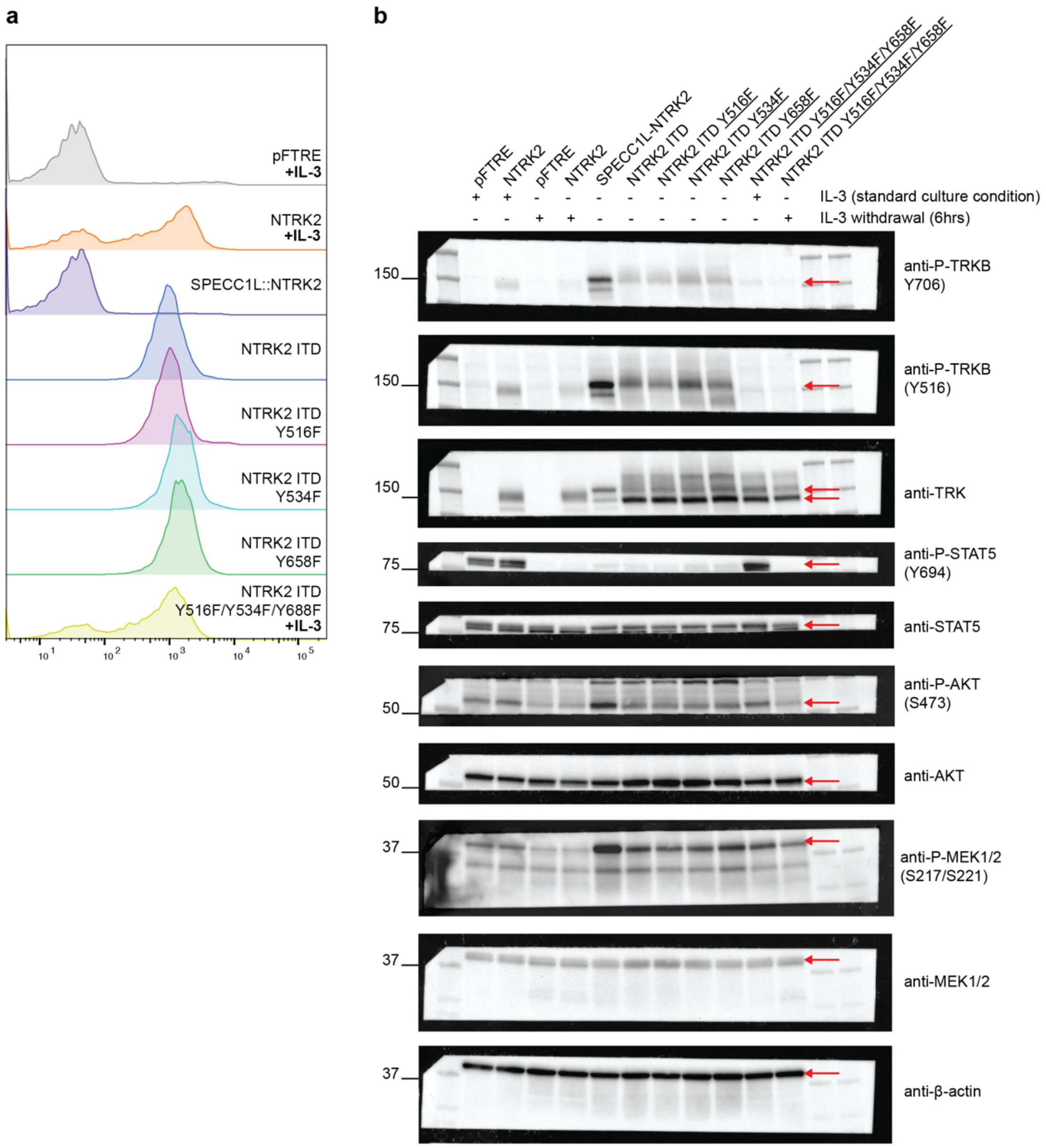

Supplementary Figure 12. NTRK2 ITD triple mutant was insufficient to transform one biologically independent cell line

a) Cell surface expression of TRKB in a single experiment where the NTRK2 ITD triple mutant was not transforming. b) Western blots of analysis of TRKB and downstream signaling pathway activation in Ba/F3 cells not transformed by the NTRK2 ITD triple mutant. Analysis was performed on pFTRE, NTRK2 and NTRK2 ITD Y516F/Y534F/Y658F Ba/F3 cells in either the presence of IL-3 or following 6-hour IL-3 withdrawal and on transformed cell lines for all other

lines (no IL-3). The locations of molecular weight markers (kDa) are indicated on the left and location of the target protein is indicated by the red arrows. Chemiluminescent images are merged with colorimetric images that were sequentially captured to determine target protein size against the molecular weight marker, using Image Lab 6.1 software (Bio-Rad Laboratories).

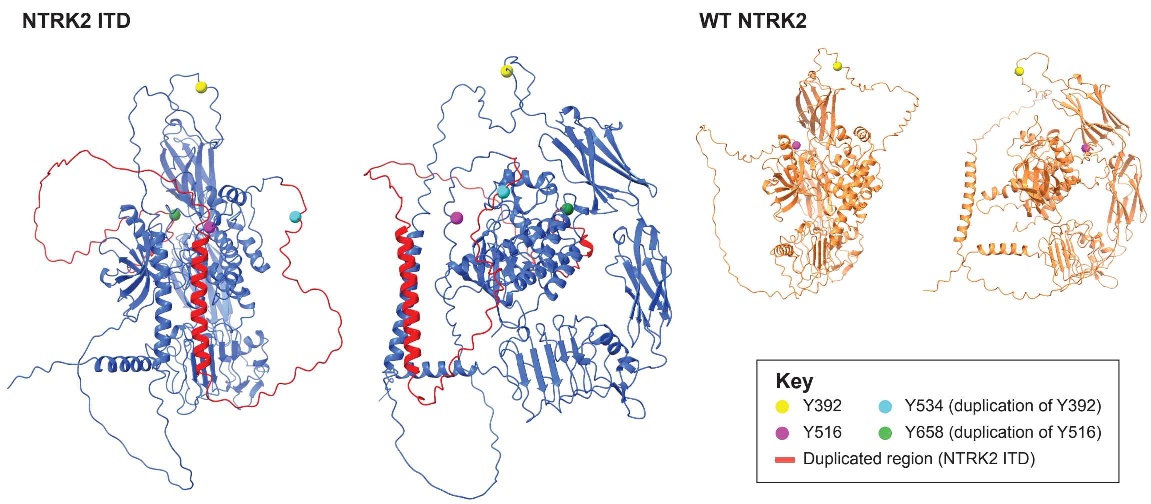

Supplementary Figure 13. Predicted NTRK2 ITD and WT NTRK2 protein structures

Predicted NTRK2 ITD (blue,) and WT NTRK2 (orange) protein structures, generated with ESMfold and visualized using ChimeraX-1.6.1. Tyrosine (Y) residues of interest and the duplicated region of the ITD are labelled according to the key.
